# Supporting mental health of healthcare staff: health economics evaluation of costs and clinical outcomes of a specialised clinic for healthcare workers

**DOI:** 10.1192/j.eurpsy.2025.321

**Published:** 2025-08-26

**Authors:** M. Kaser, K. Puri Sudhir, A. Wagner, S. Said, T. Karadaki, C. Walsh, R. Cameron

**Affiliations:** 1 Staff Mental Health Service, Cambridgeshire and Peterborough NHS Foundation Trust; 2 Department of Psychiatry, University of Cambridge, Cambridge; 3 Health Economics Group, University of East Anglia, Norwich; 4 Psychological Medicine, Cambridgeshire and Peterborough NHS Foundation Trust, Cambridge, United Kingdom

## Abstract

**Introduction:**

Healthcare workers globally face an elevated risk of mental health conditions. Despite the critical need for mental health support, access remains inconsistent, hindered by both systemic gaps and barriers like stigma and regulatory constraints. Recognizing this unmet need, we established the Staff Mental Health Service (SMHS) in Cambridgeshire, UK, in September 2020. SMHS offers prompt assessments and treatments for healthcare workers across various professional roles.

**Objectives:**

To assess costs, clinical outcomes and waiting times associated with treatment at the SMHS compared to NHS Talking Therapies (TT), a nationally-delivered primary care mental health service.

**Methods:**

A cost-consequence analysis comparing costs and clinical outcomes for a hypothetical cohort of patients (N=1,000) between two options: (1) treatment either at SMHS or TT ; and (2) treatment at TT only. SMHS costs and outcomes were collected from routinely collected patient-level data, while those for TT were informed by published literature. Only costs borne by the health system were assessed; and model outcomes were compared for one treatment period (i.e., from treatment start to discharge).  Primary model outcomes included average total treatment costs (GBP - £); and mean score changes on the Patient Health Questionnaire (PHQ-9) and Generalized Anxiety Disorder Assessment (GAD-7) measures for symptoms of depression and anxiety, respectively. Uncertainty in model parameters was explored using deterministic and probabilistic sensitivity analyses. Mean waiting times from referral to initial assessment and first treatment appointment for the SMHS and TT were analysed independently.

**Results:**

Average total treatment costs per patient for the option consisting of treatment at either SMHS or TT was £825 less costly than the option consisting of treatment only at TT (£617 vs £1,442). Main drivers of cost difference included the notably higher cost and average number of appointments associated with treatment at TT. Treatment at either SMHS or TT also led to better patient outcomes on both the PHQ-9 (mean score reductions of 7.2 vs 6.1) and GAD-7 (mean score reductions of 6.3 vs 4.8) scales. Mean waiting times (days) for treatment at SMHS were considerably shorter than at TT: referral to initial assessment (19 vs 25 days); and referral to first treatment appointment (28 vs 53 days).

**Conclusions:**

Preliminary results showed that a specialised mental health service for healthcare workers was associated with lower costs, better patient outcomes, and shorter waiting times compared to standard treatment options.

**Disclosure of Interest:**

None Declared

